# A Rare Hemorrhagic, Orange-Colored Ascites, Challenging Traditional Ascitic Fluid Analysis

**DOI:** 10.1177/23247096221150630

**Published:** 2023-01-24

**Authors:** Huma Quanungo, Huda Quanungo, Elena Naderzad, Frederick Venter, Elaine Deemer, Greti Petersen, Alan Ragland

**Affiliations:** 1 University of California, Los Angeles, USA; 2 Kern Medical, Bakersfield, California, USA

**Keywords:** ascites, orange, portal hypertension, spontaneous bacterial peritonitis, serum ascites albumin gradient, SAAG, abdominal paracentesis

## Abstract

Analysis of ascitic fluid can offer useful information in developing and supporting a differential diagnosis. As one of the most prevalent complications in patients with cirrhosis, ascitic fluid aids in differentiating a benign condition from malignancy. Both the gross appearance of the ascitic fluid, along with fluid analysis, play a major role in diagnosis. Here, we discuss a patient with liver cirrhosis, esophageal varices, hepatitis C, and alcohol abuse, who had a paracentesis performed, which revealed a turbid, viscous, orange-colored ascitic fluid that has not been documented in literature. Ascitic fluid is routinely analyzed based on gross appearance, cell count, and serum ascites albumin gradient (SAAG) score. An appearance of turbidity or cloudiness has commonly suggested an inflammatory process. In our case, fluid analysis revealed a red blood cell count of 24 250/mcL, further suggesting inflammation. However, it also revealed an insignificant number of inflammatory cells, with a total nucleated cell count of 14/mcL. This rich-orange color has posed a challenge in classification and diagnosis of the underlying cause of ascites, with one classification system suggesting inflammation, while another suggesting portal hypertension. Furthermore, we have traditionally relied on the SAAG score to aid in determining portal hypertension as an underlying cause of ascites. With a 96.7% accuracy rate, the SAAG score incorrectly diagnosed portal hypertension in this patient. In this article, we aim to explore how this rare, orange-colored ascitic fluid has challenged the traditional classification system of ascites.

## Introduction

Abdominal ascites is a pathologic collection of peritoneal fluid seen in a variety of diseases including hepatic disease, heart failure, kidney disease, malignancy, infection, and more.^1^ Determining the cause of newly presenting ascites is crucial in diagnosis, treatment, and decreasing mortality rates. Performing abdominal paracentesis for ascitic fluid analysis is an essential factor in determining the underlying cause of ascites.^2^ Various properties are used to evaluate ascitic fluid such as gross appearance, cell count, and differential, serum ascites albumin gradient (SAAG), enzymes, macromolecules, culture and gram stain, and tumor markers.^3^ Both laboratory data and clinical findings are used in conjunction to establish a diagnosis to provide treatment and disease prognosis. Traditionally, once all these parameters are analyzed, the underlying cause of ascites usually becomes apparent, helping direct medical therapy.

## Case Presentation

A 56-year-old Hispanic woman with a history of alcoholic cirrhosis, grade 3 esophageal varices status-post banding in 2017, and untreated hepatitis C, presented with abdominal distension and fatigue for 1 month. She presented multiple times to the emergency room for abdominal discomfort due to ascites, requiring paracentesis. In addition, she was noted to have anemia on these occasions and required blood transfusions. She never had been diagnosed with spontaneous bacterial peritonitis (SBP), nor did she have any recent gastrointestinal (GI) bleeding. She presented with abdominal pain secondary to ascitic fluid accumulation. On physical examination, the abdomen was distended with mild tenderness to palpation diffusely. Shifting dullness, caput medusa, and spider angiomata were noted. Her skin was jaundiced with associated scleral icterus. She had bilateral lower extremity pitting edema up to the knees. Laboratory investigations at that time revealed: hemoglobin of 6.0g/dL, platelets of 115 000/mcl, serum albumin 1.8 g/dL, direct bilirubin of 1.8 mg/dL, total bilirubin of 4.8 mg/dL, and total protein of 8.3g/dL. Coagulation studies showed prothrombin time (PT) of 20.6 seconds, partial thromboplastin time (PTT) of 2.1 seconds, and an international normalized ratio (INR) of 1.81. Vitamin D level was 125 ng/mL and vitamin B12 level was 2451 pg/mL. Chest X-ray and electrocardiogram were unremarkable. On computed tomography scan of the pelvis, a moderate amount of ascites was associated with stigmata of cirrhosis, and portal venous hypertension was manifested by severe splenomegaly.

**Image 1.**
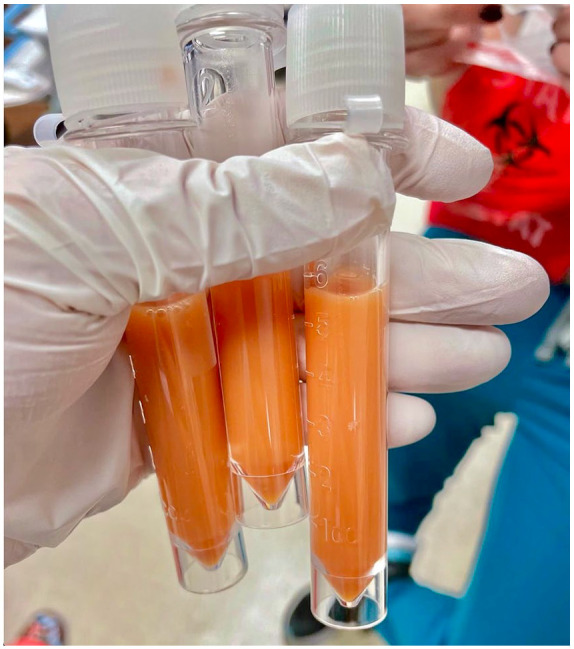
Abdominal paracentesis revealing turbid, viscous orange-colored ascitic fluid.

An abdominal paracentesis was performed and revealed a unique bright orange-colored, turbid, and viscous ascites (Image 1) with total polymorphonuclear (PMN) count of 14/mcL, red blood cell (RBC) count of 24 250/mcL, and differential of 11% neutrophils, 55% lymphocytes, 14% monocytes, and 20% macrophages. In addition, analysis of the ascitic fluid showed an albumin level of 0.7 g/dL, lactate dehydrogenase (LDH) level of 118 U/L, pH level 7.70, and protein level of 2500 mg/dL. Spontaneous bacterial peritonitis was ruled out based on PMN count <250/mm^3^. Serum ascites albumin gradient score was calculated as 1.1 with 96.7% accuracy of correctly predicting that the ascites was due to portal hypertension. Her Child-Pugh score was 12, categorizing her as Child Class C with a life expectancy of 1 to 3 years with abdominal surgery and perioperative mortality of 82%. Her Model for End-Stage Liver Disease (MELD) score was 23 points, with a 19.6% estimated 3-month mortality.Body fluid culture with gram stain was negative. Hepatocellular carcinoma was ruled out, as evidenced by her normal alpha-fetoprotein (AFP) level and lack of imaging findings. She was subsequently started on intravenous pantoprazole, octreotide, furosemide, spironolactone, and lactulose therapy.

Given the above analyses, ascites cytology opens a discussion about the possibility of a non-inflammatory, hemorrhagic process underlying her disease. The clinical picture of this patient and high protein count pointed us toward an inflammatory pathology initially; however, this does not appear to be the case, as evidenced by her cellular profile showing minimal PMNs. The significantly elevated RBC count does not appear to be from an acute process, but more likely a long-term process. This can be supported by the increased total protein concentration, which will often be elevated in a long-term process. On one hand, we have an SAAG score that pointed us toward the diagnosis of portal hypertension. On the other hand, we have a significantly elevated protein count that cannot be explained by portal hypertension alone, and therefore, must be attributed to another underlying process such as a spontaneous hemorrhagic, noninflammatory, yet exudative pathology.

## Discussion

Ascites is defined as the disruption in intravascular and extravascular fluid spaces, resulting in pathologic accumulation of >25 mL of fluid in the peritoneal cavity.^4^ This can occur due to numerous pathologies including, but not limited to, portal hypertension, hypoalbuminemia, and peritoneal disease. We will focus our discussion on the development of portal hypertension in the setting of cirrhosis, given that our patient presented with alcoholic cirrhosis. In these patients, portal hypertension is a prerequisite for the development of cirrhotic ascites, serving as a landmark in the natural history of cirrhosis with a poor prognosis of 50% mortality within 3 years.^1^ In other words, ascites does not occur in cirrhotic patients without portal hypertension. The mechanism of portal hypertension continues to be studied. It begins with arterial splanchnic vasodilation, which can be attributed to increased nitric oxide production. Another contributing factor is the degree of structural changes within the hepatocytes due to fibrosis, nodule formation, and vascular occlusion (depending on the degree of cirrhosis).^5^ In turn, the body undergoes changes in hemodynamics to maintain blood flow by the renin-angiotensin-aldosterone system (RAAS) activation, anti-diuretic hormone (ADH) secretion, sympathetic nervous system regulation, and increased production of endogenous vasoconstrictors. Vasoconstrictors contribute to the dynamic changes seen in the liver by activating the contraction of hepatic stellate cells and myofibroblasts. Renal sodium excretion becomes impaired, leading to a positive sodium balance and water retention. Hepatic vascular resistance then increases due to expansion of extracellular fluid, causing hydrostatic forces within hepatic sinusoids, typically greater than 12 mm Hg. These hydrostatic forces cause fluid to transude into the peritoneal space, beginning the cycle of portal hypertension. As portal hypertension worsens, there is a continued increase in local release of splanchnic vasodilators leading to systemic hypotension, plasma volume expansion, and increased cardiac output.^6^

One of the most common complications of portal hypertension is spontaneous bacterial peritonitis. Although the pathophysiology is still being investigated, it is proposed that SBP occurs due to interaction between changes in gut microbiome, intestinal permeability, and immune dysfunction.^7^ Pathogenic growth of gut bacteria in patients with cirrhosis occurs due to slowed mucosal blood flow, thereby promoting bacterial overgrowth and translocation. An ascites PMN count ≥250/mm^3^ indicates peritonitis that may be due to SBP or secondary causes. Risk factors include upper GI bleed and low ascitic protein concentration.^2^

Ascites is routinely analyzed via fluid samples based on appearance of the fluid, total protein concentration, SAAG score, cell count, and total protein concentration.^3^ Gross appearance of ascites fluid can be useful in providing preliminary signs related to the cause of an underlying disease. Clear or straw-colored ascitic fluid is often associated with uncomplicated ascites in the setting of cirrhosis. Turbid or cloudy ascites is associated with infected fluid as seen in bacterial infection or peritonitis (SBP). Milky or chylous ascites is defined as a fluid rich in triglycerides and proteins, which is often seen in malignancy, tuberculosis, or parasitic disease. Hemorrhagic or bloody ascites, which is defined as ascitic fluid RBC count >10 000/μL, may indicate a “traumatic tap” during paracentesis, or malignancy such as hepatocellular carcinoma.

We begin our classification of ascites in this patient by examining the gross appearance of fluid extracted by abdominal paracentesis. Our patient presented with an orange-colored, turbid ascites, featuring an unfamiliar appearance that is not documented in literature. The color and fluid properties of the ascites is so unique, it does not fit into the traditional classification system that has categorized ascites by its gross appearance. Fluid analysis revealed an RBC count of 24 250/mcL, which suggests an inflammatory process is at play here. However, appearance as a predictor of underlying disease is challenged by the cell count and differential in the ascitic fluid. Furthermore, ascitic fluid RBC count, in addition to physical exam findings and bloody ascites strongly suggest hemorrhagic ascites. Hepatocellular carcinoma would be the strongest differential diagnosis given her findings; however, even this suspicion was minimized due to an AFP level of 6.5 ng/mL and negative imaging studies. AFP levels have a specificity of 80% in detecting hepatocellular carcinoma.^8^ This causes us to move on to investigating the cell count and differential, which notably has the greatest diagnostic power for ascitic fluid.

We next consider the white blood cell (WBC) and PMN leukocyte count to exclude SBP and investigate other potential causes of ascitic fluid. Based on the unique cell profile found in our orange-colored ascites of low WBC, high protein, and high RBC, we are faced with a challenge in further classifying this fluid. The total protein concentration was formerly used to classify ascitic fluid as exudative or transudative, with a diagnostic criterion of >2.5g/dL to be exudative.^3^ Total protein concentration has since been widely replaced with SAAG but can still be valuable in differentiating pathologies of high-protein ascites and low-protein ascites. Our team originally believed the diagnosis was portal hypertension, which was contradicted by the high protein count we found in our fluid. The typical finding in cirrhosis, and what we had expected to find, is a low-protein ascitic fluid. High-protein ascites, as in our patient, is usually explained by other processes such as congestive heart failure, constrictive pericarditis, peritoneal carcinomatosis, tuberculosis (TB), and Budd-Chiari syndrome, all of which were excluded upon further evaluation.^3^ At this point, we seek to find another source for the elevated total protein count found in our ascitic fluid, which we can now definitively state was not a product of portal hypertension but likely some other underlying pathophysiology that has not revealed itself.

To further classify the ascitic fluid, the SAAG score was calculated. The SAAG score is a diagnostic calculation tool used in the evaluation of ascites. It is based on the difference between the ascitic fluid albumin level and serum albumin level, reflecting the degree of portal hypertension, as well as prognosis in a patient with cirrhosis.^3^ Prior to the SAAG score, ascitic fluid was classified into transudate versus exudate based on ascitic fluid total protein (AFTP). The AFTP classified ascites correctly only 55.6% of the time, and thus was replaced with the SAAG score due to greater diagnostic accuracy.^9^ A SAAG score >1.1 g/dL indicates that a patient has portal hypertension, most notably with an accuracy of 96.7%.^1^ In our patient, serum albumin level is 1.8 mg/dL and the ascites fluid albumin level is 0.7 mg/dL resulting in an SAAG score of 1.1, which is nondiagnostic for portal hypertension as the cause of ascites. Depending on the source, the SAAG score parameters can have differing cutoff values. Based on the Annals of Internal Medicine, an SAAG score of ≥1.1 is indicative of portal hypertension, whereas other sources designate >1.1 or <1.1 as their diagnostic parameters.^9^ The inconsistent definitions lend to the variability of application of the SAAG score.

**Image 2 and 3.**
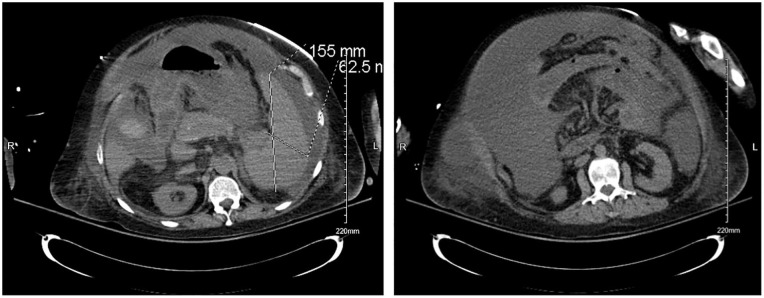
Computed tomography revealing moderate amount of ascites with stigmata of cirrhosis and portal venous hypertension.

In contrast to the inconclusive SAAG score results, computed tomography of the pelvis and abdomen (Images 2 and 3) in this patient revealed the presence of portal venous hypertension. Furthermore, our patient had cirrhosis, indicating the underlying mechanism of portal hypertension is in fact present, confirmed with imaging. Based on these criteria, our patient has fallen in the 3% in which the SAAG score is not an accurate diagnostic tool, further complicating the classification of her ascites.^10^

## Conclusion

Physicians have traditionally used the SAAG score to help differentiate between abdominal ascites being due to portal hypertension versus inflammatory processes with 96.7% accuracy. Our case highlights the misdiagnosed 3% of patients with ascites. The prevalence of liver cirrhosis in the United States was 633323 adults in 2015.^11^ Up to 50% of decompensated cirrhotic patients develop ascites as an associated complication.^12^ Approximately 3% of the population with ascites fluid in 2015 would estimate up to 9499 patients who are unable to be evaluated by the SAAG score. Our case is an example of this fact, where using the SAAG score suggested causes of ascites fluid that contradicted our patient’s laboratory findings. Thus, in clinical settings that do not conform with traditional diagnostic tools, physicians cannot rely on these measures to help guide diagnoses. Our team suggests that there needs to be more research into quick score calculations used to classify ascitic fluid.
